# Metal-organic framework-modulated Fe_3_O_4_ composite au nanoparticles for antibacterial wound healing via synergistic peroxidase-like nanozymatic catalysis

**DOI:** 10.1186/s12951-023-02186-6

**Published:** 2023-11-15

**Authors:** Chuan Liu, Xuanping Zhao, Zichao Wang, Yingyuan Zhao, Ruifang Li, Xuyang Chen, Hong Chen, Mengna Wan, Xueqin Wang

**Affiliations:** 1 College of Bioengineering, Henan University of Technology, Zhengzhou, 450001 Henan China; 2 Key Laboratory of Functional Molecules for Biomedical Research, Henan University of Technology, Zhengzhou, 450001 Henan China

**Keywords:** Hybrid nanozyme, Peroxidase, Metal-organic framework, Anti-microbial therapy, Infected wound healing

## Abstract

Bacterial wound infections are a serious threat due to the emergence of antibiotic resistance. Herein, we report an innovative hybrid nanozyme independent of antibiotics for antimicrobial wound healing. The hybrid nanozymes are fabricated from ultra-small Au NPs via in-situ growth on metal-organic framework (MOF)-stabilised Fe_3_O_4_ NPs (Fe_3_O_4_@MOF@Au NPs, FMA NPs). The fabricated hybrid nanozymes displayed synergistic peroxidase (POD)-like activities. It showed a remarkable level of hydroxyl radicals (·OH) in the presence of a low dose of H_2_O_2_ (0.97 mM). Further, the hybrid FMA nanozymes exhibited excellent biocompatibility and favourable antibacterial effects against both Gram-negative *(Escherichia coli)* and Gram-positive (*Staphylococcus aureus*) bacteria. The animal experiments indicated that the hybrid nanozymes promoted wound repair with adequate biosafety. Thus, the well-designed hybrid nanozymes represent a potential strategy for healing bacterial wound infections, without any toxic side effects, suggesting possible applications in antimicrobial therapy.

**Supplementary Information:**

The online version contains supplementary material available at 10.1186/s12951-023-02186-6.

## Introduction

Bacterial infection is one of the most serious risk factors threating public health worldwide [1]. Antibiotics are traditionally used to treat bacterial infections [2]. However, the prolonged overuse and misuse of antibiotics has led to antibiotic inactivation and the emergence of multidrug-resistant bacteria [3, 4]. According to the World Health Organization (WHO), infections caused by antibiotic-resistant bacteria threaten the life of approximately 700,000 humans worldwide every year, and the number is expected to rise to 10 million by 2050, more than the number of cases attributed to tumours currently [5, 6]. Therefore, it is imperative to develop innovative and effective antimicrobial agents against pathogenic bacteria.

The emergence and rapid development of antibacterial nanomaterials such as silver nanoparticles (NPs), zinc NPs, and copper NPs have attracted tremendous attention due to their superior antibacterial performance [7–9]. However, despite their enormous potency, antibacterial nanomaterials have low biosafety and therefore cannot be used in clinical applications. Thus, it is critical to develop nanomaterial-based antibacterial agents with strong biosafety. To eliminate the growing threat of drug-resistant bacterial infections, extensive efforts have been devoted to develop effective nanomaterials as antimicrobial agents [10–12].

Nanozymes, which are nanomaterials with natural enzyme-mimetic catalytic performance, have been used extensively in biomarker detection [13], cancer diagnosis and therapy [14], biosensing [15], and food analysis due to their low cost, high stability, easy storage, tunable catalytic activities, and easy functional modification compared with natural enzymes [16, 17]. Since Yan et al. first reported that magnetite (Fe_3_O_4_) NPs exhibit reaction kinetics and catalytic mechanisms similar to those of natural horseradish peroxidase [18], many nanomaterials with different composition and similar enzymatic properties have been developed [19, 20]. Nanozymes have emerged as new tools to defeat bacterial infection, thereby attracting increased attention for antibacterial applications [21–23].

Many monometallic nanozymes, such as Tb_4_O_7_ NPs, silica-loaded AuNPs, and Pd NPs, exhibit bactericidal effects. However, due to their weak catalytic activity, high concentrations are required to achieve optimal antimicrobial effect. Hence, polymetallic nanozymes maybe a promising strategy to achieve highly catalytic performance [24–26]. Hydrogen peroxide (H_2_O_2_) is a common antibacterial agent using for sterilisation and disinfection, which can effectively destroy bacterial proteins and DNA leading to cell lysis and death [27, 28]. However, its antibacterial ability depends on the concentration of H_2_O_2_, which is usually needed in high concentrations for bactericidal effects [29]. Nevertheless, high concentrations of H_2_O_2_ can corrode healthy skin tissues and exacerbate pathological inflammation, and even damage the central nervous system in humans [30, 31], peroxidase (POD)-like nanomaterials have been developed to catalyse substrates to generate extremely toxic ·OH under low levels of H_2_O_2_ for antibacterial treatment [32–34]. Therefore, enhancing the POD-mimetic activity of nanomaterials represents an effective antibacterial strategy.

For more than a decade, Fe_3_O_4_ NPs with intrinsic POD-mimetic performance have been utilised in biomedical applications. However, bare Fe_3_O_4_ NPs are highly unstable and easily aggregate due to the large surface energy, which greatly reduced their catalytic performance. A coating layer of mesoporous materials has been reported to prevent the agglomeration of Fe_3_O_4_ NPs and further improved their stability. Additionally, the synergetic interactions between Fe_3_O_4_ NPs and another catalyst can enhance the enzyme-mimetic activity. Therefore, the fabrication and stabilisation of a hybrid nanozyme based on Fe_3_O_4_ NPs has been shown to improve antibacterial treatment. Among the various coating layers available, metal–organic frameworks (MOFs) with high specific surface area, high porosity, tunable size, accessible active sites, and significant capacity to accommodate guest molecules by anchoring them in proper position, have attracted substantial interest [35–37]. A variety of modified MOFs have been synthesised by introducing functional groups into organic ligands, such as porous isoreticular metal-organic frameworks (IRMOF-3), which improved their aperture size and surface properties. Additionally, catalysts developed by incorporating metal nanoparticles into MOFs facilitated the study of heterogeneous reactions.

**Scheme 1:**
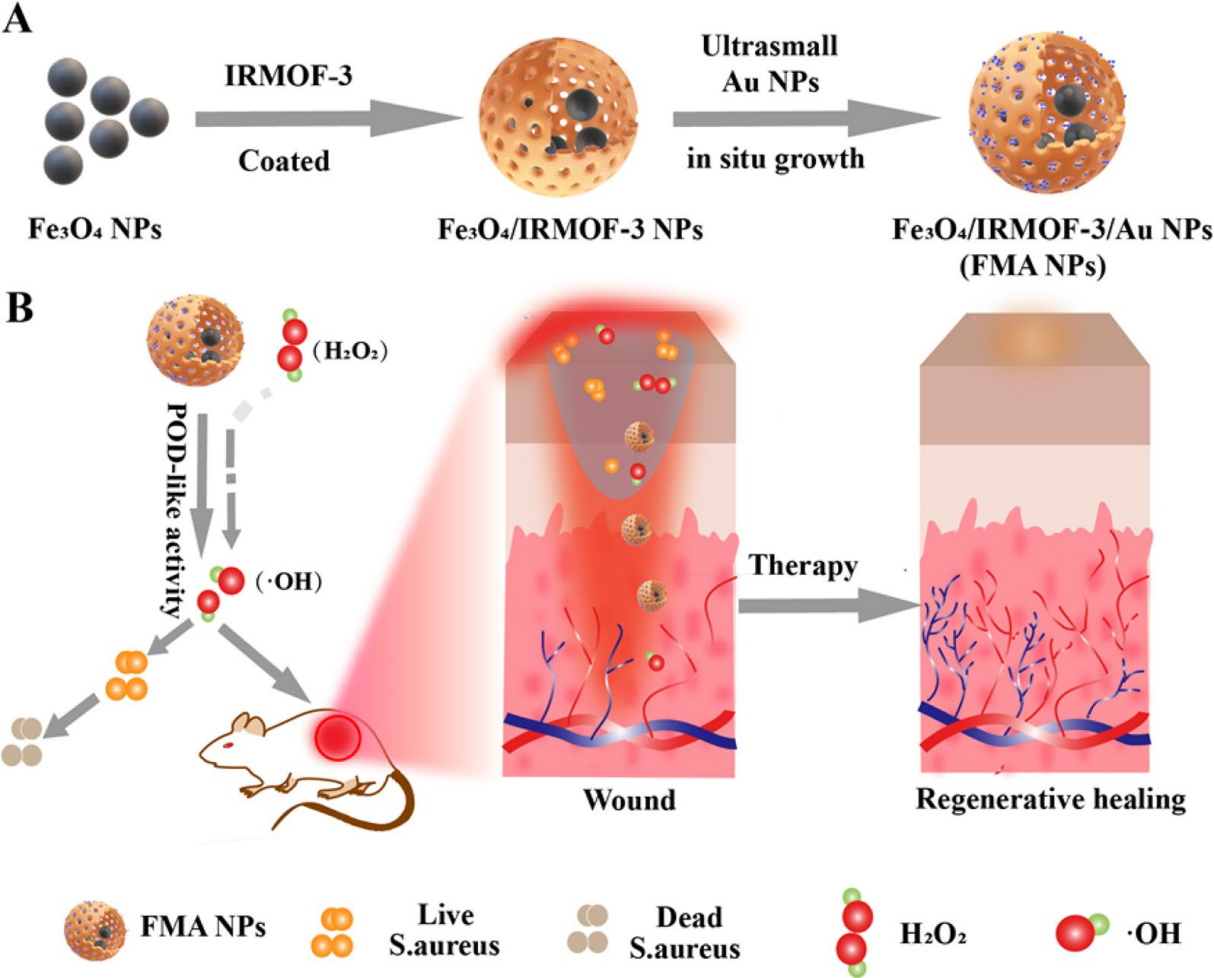
** A** Schematic diagram of the preparation of IRMOF-3-stabilised FMA hybrid nanozymes. **B** Mechanism of antibacterial wound healing based on high POD-like activities of FMA nanozymes in vivo

Herein, we designed an innovative nanocatalytic antibacterial strategy based on MOF-modulated bimetallic hybrid nanozymes for infected wound healing without employing any antibiotic drugs (Scheme 1). Magnetic Fe_3_O_4_ NPs were initially prepared via chemical coprecipitation. Porous IRMOF-3 were then used for surface decoration of Fe_3_O_4_ NPs as linkers, resulting in functional modification. Ultrasmall Au NPs were successively integrated into IRMOF-3 to construct heterogenous bimetallic composite nanozymes, i.e., Fe_3_O_4_@MOF@Au NPs (FMA NPs). In principle, these FMA nanocatalysts induce an ROS-mediated antimicrobial mechanism in bacteria, thereby significantly minimising bacterial growth. This well-designed FMA nanozymes have prominent advantages as follows: (i) The synergistically enhanced POD-like activities via cascade reaction of Au and Fe_3_O_4_ NPs greatly accelerating catalytic reaction rate and further improving the antibacterial efficiency; ii) the improved dispersion stability, reduced oxidation and biosafety by surface encapsulation of Fe_3_O_4_ NPs using MOF nanoshell layers; (iii) the bimetallic nanozymes combined with the low concentration of H_2_O_2_ (0.97 µM) for achieving excellent therapeutic effects.

Therefore, the FMA hybrid nanozymes were expected to exhibit improved enzymatic activity and effective antibiotic treatment.

## Materials and methods

### Materials

All experimental materials and methods of characterisation are described in detail in the Supporting Information.

### Animals

Approximately 5-week-old Kunming (KM) mice (female, 18∼20 g) were obtained from the Animal Experiment Center of Zhengzhou University (Zhengzhou, China) and fed under specific pathogen-free (SPF) conditions. Animal experiments were approved by the Animal Care and Use Committee of the School of Biological Engineering, Henan University of Technology (Ethics Approval Code: Haut202110 − 5).

### Synthesis of MOF-modulated Fe_3_O_4_ NPs

Superparamagnetic Fe_3_O_4_ NPs were synthesised via chemical co-precipitation [38, 39]. Additional details provided in Supplementary Information. The IRMOF-3-modulated Fe_3_O_4_ NPs were synthesised using a high-pressure one-pot method [40]. Briefly, 10 mg of Fe_3_O_4_ NPs were mixed with 5 mL of anhydrous ethanol and 5 mL of N-N dimethylformamide (DMF), followed by treatment with 200 mg of polyvinylpyrrolidone (PVP) as a dispersant under ultrasonication for 20 min. Simultaneously, 67 mg of zinc nitrate and 16 mg of 2-aminoterephthalic acid were dissolved in 4 mL of DMF to form a mixture. Subsequently, the two solutions were combined and sonicated for 10 min in an ultrasonic emulsion disperser. The mixture was transferred to an autoclave and heated for 4 h at 100 °C. Brown products were acquired via magnetic decantation under a magnetic field, and the residue was dispersed again into 20 mL of DMF. The products were washed with deionised water (DI) six times to obtain Fe_3_O_4_@ IRMOF-3 NPs.

### Loaded ultrasmall AuNPs

Fe_3_O_4_@ IRMOF-3 NPs (80 mg), prepared as above, were dispersed into 40 mL of DI, followed by 20 min of sonication. Next, 4 mL of HAuCl_4_ solution (20 mM) was added and sonicated for 20 min, followed by 10 mL of freshly frozen prepared 0.1 M NaBH_4_. The suspension was stirred at 120 rpm for 1 h. The fabricated Fe_3_O_4_@MOF@Au NPs (FMA NPs) were washed three times using DI water for further use.

### Evaluation of POD-like catalytic performance

The POD-like catalytic performance of the as-prepared Fe_3_O_4_ NPs, Fe_3_O_4_@MOF NPs, and FMA NPs was investigated using 3, 3’, 5, 5’-tetramethylbenzidine (TMB) as the chromogenic substrate. Next, 50 µL of various nanocomposites (1 mg/mL) were added to 500 µL of NaAc-HAc buffer solution consisting of 20 µL H_2_O_2_ (30%) and 10 µL TMB (10 mg/mL), respectively. The absorbance at 652 nm was measured with a microplate spectrophotometer (Bio Tek Instrument Inc. USA). Catalytic activities of nanozyme mimetics or natural enzymes were controlled by reaction pH, reaction temperature and reaction time [41]. Therefore, the relative catalytic activities of Fe_3_O_4_, Fe_3_O_4_@MOF, and FMA NPs under different pH levels (2.8 ~ 8.4), temperature (20 ~ 60 °C), and reaction time (0 ~ 30 min) were analysed. The natural POD was used as a positive control.

### Kinetic analysis

The analysis of steady-state kinetics is described in detail in the Supporting Information.

### Study of •OH generation

The production of ·OH in the presence of the spin trap 5, 5-dimethyl-1-pyrroline-N-oxide (DMPO) was first analysed qualitatively via electron spin resonance (ESR) spectroscopy using 400 µL of FMA NPs (400 µg/mL) and 20 µL of H_2_O_2_ (0.2 M) after 15 min. Quantitative analysis of ·OH production was performed using a microplate.

### Evaluation of hemocompatibility

Fresh blood (5 mL) acquired from KM mice was dispersed in 0.9% saline solution, and centrifuged at 2500 × g for 5 min three times. The supernatant was then discarded. The acquired red blood cells (RBCs) were washed with 0.9% saline solution, and diluted with an appropriate amount of 0.9% saline solution to prepare 2% RBC suspension. Next, 1 mL RBC suspension was added to solutions containing varying concentrations (50, 100, 200, 400 µg/mL) of FMA NPs. Saline solution (0.9%) and DI water served as negative and positive controls. respectively. The resulting products were centrifuged at 3000 rpm for 5 min after 4 h of stabilisation at room temperature (RT). The absorbance of 100 µL of each supernatant was measured at 540 nm to calculate the hemolysis rate as follows.

$$\it \it {\text{Hemolysis rate }}(\% ) = \frac{{OD_{c} - OD_{b} }}{{OD_{a} - OD_{b} }}\, \times \,100\%$$

where *OD*_*a*_ is the absorbance of the positive control; *OD*_*b*_ is the absorbance of the negative control; and *OD*_*c*_ denotes the absorbance of the samples at 540 nm.

### Cell and bacterial culture

Normal liver L-02 cells and human umbilical vein endothelial cells (HUVECs), acquired from the Shanghai Cell Bank of the Chinese Academy of Sciences (Shanghai, China), were cultured in DMEM medium containing 10% fetal bovine serum, 1% penicillin (100 U/mL), and 1% streptomycin (100 µg/mL), in a cell incubator at 5% CO_2_ and 37 °C.

Gram-positive *S. aureus*, and the Gram-negative *E. coli* bacteria, obtained from China General Microbiological Culture Collection Center were cultured in Luria-Bertani (LB) liquid medium. They were incubated at 37 °C.

### Evaluation of in vitro cytotoxicity

Normal liver L-02 cells and HUVECs were used to assess the cytotoxicity of FMA NPs. The L-02 cells and HUVECs were treated for 24 h with various concentrations of FMA NPs. Subsequently, 20 µL of CCK-8 was added to each micro well and cultured for another 4 h in the dark. The absorbance at 450 nm was measured using a multifunctional microplate reading instrument to evaluate the toxic effects of FMA NPs.

### Analysis of cell viability

The cell viabilities of treated liver L-02 cells and HUVECs were evaluated via PI and FDA double-staining assay, respectively. Cells were treated with different formulations for 24 h. The control groups were treated with serum-free medium. After washing twice with sterile PBS (pH 7.4), the treated cells were supplemented with a solution containing 100 µL PI (20 µg/mL) and 100 µL FDA (1 µg/mL). After incubation in the dark for 15 min at 37 °C, the samples were photographed under an inverted fluorescence microscope (Bio-Tek Epoch, Beijing, China).

### Analysis of antibacterial performance in vitro

We investigated the antibacterial effects of various formulations against *S. aureus* and *E. coli* using the plate count method [42, 43]. The bacteria were inoculated in a 10 mL microtube and incubated in a constant temperature shaker at 37℃ until the OD_600nm_ value of the bacterial suspension was around 0.7. The bacterial suspension was then diluted with sodium acetate buffer (pH 4.5) to a concentration of 10^7^ CFU/mL, followed by the addition of 50 µL of the bacterial suspension to various groups including control, H_2_O_2_ group (0.97µM), FMA NPs group, and the groups treated with (low dose) FMA NPs + H_2_O_2_ (200 µg/mL), and (high dose) FMA NPs + H_2_O_2_ (400 µg/mL). The minimum inhibitory concentrations (MICs) of FMA NPs were also determined under different concentrations of FMA NPs and under similar conditions to analyse the MICs of *S. aureus* and *E. coli*. Bacterial suspensions were cultured on LB agar plates and the number of CFU was calculated after 24 h of incubation at 37 °C. The bacterial inhibition rate was defined using the following equation:

$$Antibacterial\,rate\left(\%\right)=\frac{{C}_{o}-C}{{C}_{o}}\times 100\%$$

In the equation above, *C* represents is the number of colonies in different experimental groups, and *C*_*O*_ denotes the number of colonies in the control group.

### Morphology of treated bacteria

The morphology of the treated bacteria was analysed via scanning electron microscopy (SEM). Samples of *S. aureus* and *E. coli* treated with various formulations for 2 h were collected by centrifugation at 2000 rpm for 10 min, and the supernatant was discarded. The bacteria were then fixed with 2.5% glutaraldehyde at 4 °C for 12 h. Subsequently, the fixed bacteria were washed with PBS and then dehydrated for 20 min using a gradient of 30%, 50%, 70%, 80%, 90%, and 100% ethanol. After drying in a critical point drying apparatus for 90 min, the treated bacteria were sprayed with gold for 150 s and the morphological changes were observed via SEM (Hitachi, SU8020).

### Biofilm-scavenging assay

The biofilm formation and detection was evaluated as described previously with minor modification [44]. The biofilms were formed after incubation with *E. coli* (10^7^ CFU/mL) and various formulations for 24 h at 37 °C. The biofilms were carefully washed twice with sterilised PBS to remove the supernatant. The remaining biofilms were stained for 30 min with 20 µL for 30 min with 20 µL of 2% crystalline violet solution at room temperature (RT), followed by the addition of 250 µL anhydrous ethanol solution to dissolve the crystalline violet for further semi-quantitative analysis of biofilm. The OD values of the samples were detected with a microplate spectrophotometer at 590 nm. The biofilm formation was evaluated and *S. aureus* infection was detected.

To evaluate the inhibitory effects of the biofilm, a 250 µL suspension of *E. coli* (10^7^ CFU/mL) was first added to 24 microplates. The biofilm formation was detected at the bottom of the microplates after incubation for 24 h at 37 ℃. Subsequently, various groups were treated with the formulations and incubated at 37 ℃ for another 24 h. The inhibition of biofilm was measured via crystalline violet staining as described above [45].

### Live and dead bacterial fluorescence analysis

We used SYTO 9/PI double-labelling to evaluate the vitality of the treated bacteria. Live bacteria were stained with SYTO 9, displaying green fluorescence. Dead bacteria were stained with PI, displaying red fluorescence. Following treatment for 60 min at RT with various formulations, the treated *S. aureus* samples were stained in the dark for 15 min with the nucleic acid dye SYTO 9 and PI at RT. The stained bacteria were visually analysed with an inverted fluorescence microscope (Bio-Tek Epoch, Beijing, China).

### Mouse model of bacterial wound

To assess the antibacterial activity of FMA nanozymes in vivo, KM mouse models were established. The mice were first dehorned. A circular skin measuring about 6–8 mm in diameter was cut off the backs of mice with scissors. A 20 µL solution of *S aureus* (10^7^ CFU/mL) was added dropwise to the wound to create an *S. aureus*-infected wound. The infected mice were randomly divided into 5 groups and treated for 8 days using different formulations. The wounds were photographed every day. The treated mice were executed on day 9 after treatment. The skin tissues of the infected mice were obtained from each group separately, fixed using 4% formaldehyde, paraffin-embedded, and stained using hematoxylin-eosin (H&E).

### In vivo biosafety

To further evaluate the biosafety of FMA nanozymes, the major organs including heart, liver, spleen, lung, and kidney of wound mice were dissected and fixed with 4% formaldehyde to prepare paraffin sections for H&E staining.

### Statistical analysis

The methods of statistical analyses are described in the Supporting Information.

## Results and discussion

### Preparation and characterisation of FMA NPs

**Scheme 2:**
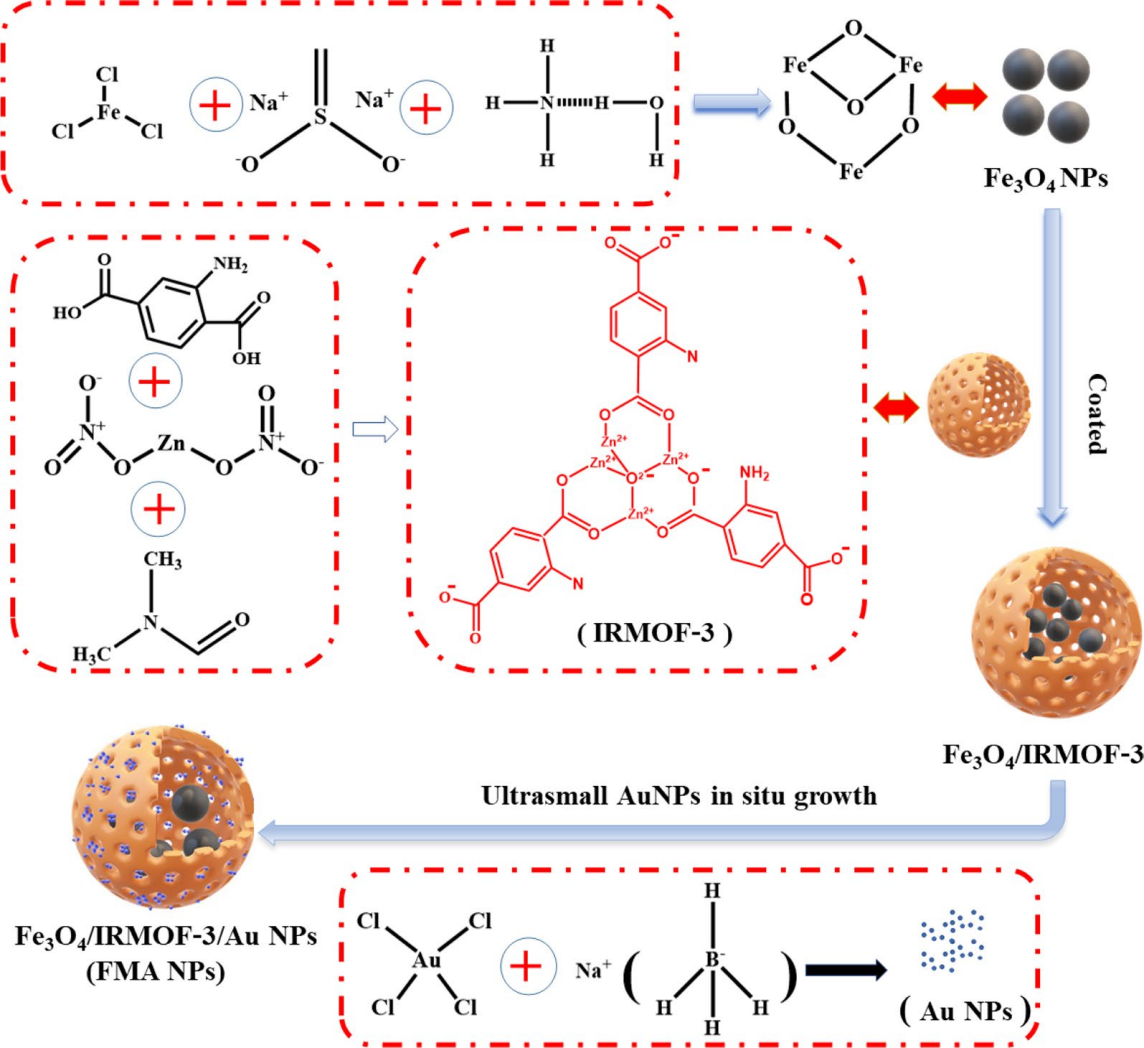
Schematic diagram of the synthesis of FMA NPs

We developed an innovative nanocatalytic antibacterial nanosystem using IRMOF-3-modulated biomimetic hybrid FMA NPs. The FMA NPs were assembled with ultrasmall Au NPs and Fe_3_O_4_ NPs displaying synergistic POD-mimicking activities for infected wound healing without using any antibiotics. Scheme 2 presents the synthetic mechanisms of hybrid FMA nanozymes: (i) preparation of superparamagnetic Fe_3_O_4_ NPs via partial reduction chemical co-precipitation; (ii) modification of Fe_3_O_4_ NPs with highly porous IRMOF-3 shells to fabricate Fe_3_O_4_@MOF NPs using the hydrothermal method; and (iii) incorporating ultrasmall Au NPs into the pores of IRMOF-3 shells in situ, which enhanced their POD activities via a cascade reaction to form flexible multiphase catalysts FMA NPs.

**Fig. 1:**
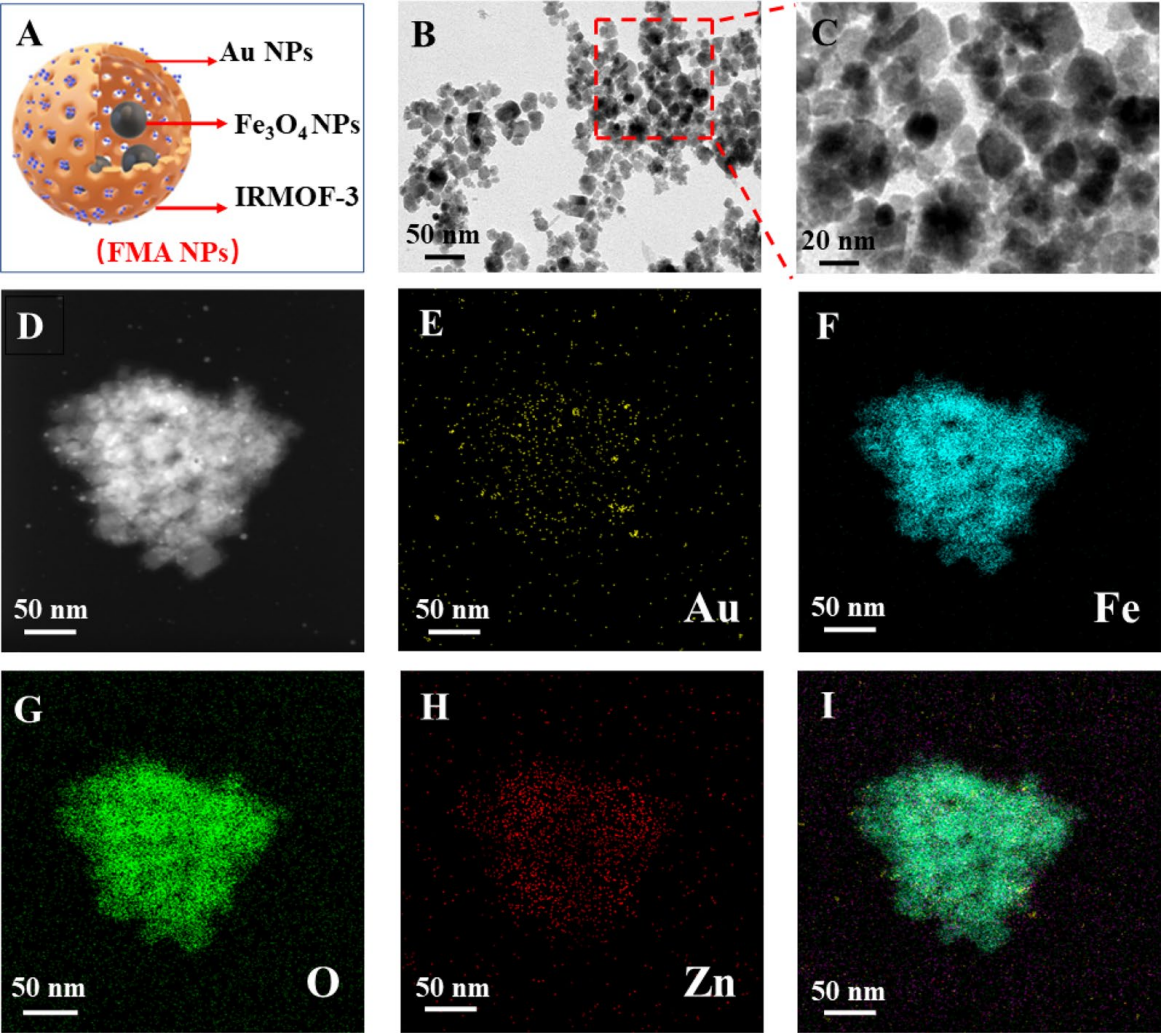
Characterisation of FMA NPs. **A** Schematic diagram of FMA NPs. **B** TEM image of FMA NPs. **C** Enlarged TEM image of FMA NPs. **D** HAADF-STEM image. **E**-**I** Elemental mapping of the corresponding regions of FMA NPs (for Au Fe, O, Zn and Merge)

**Fig. 2:**
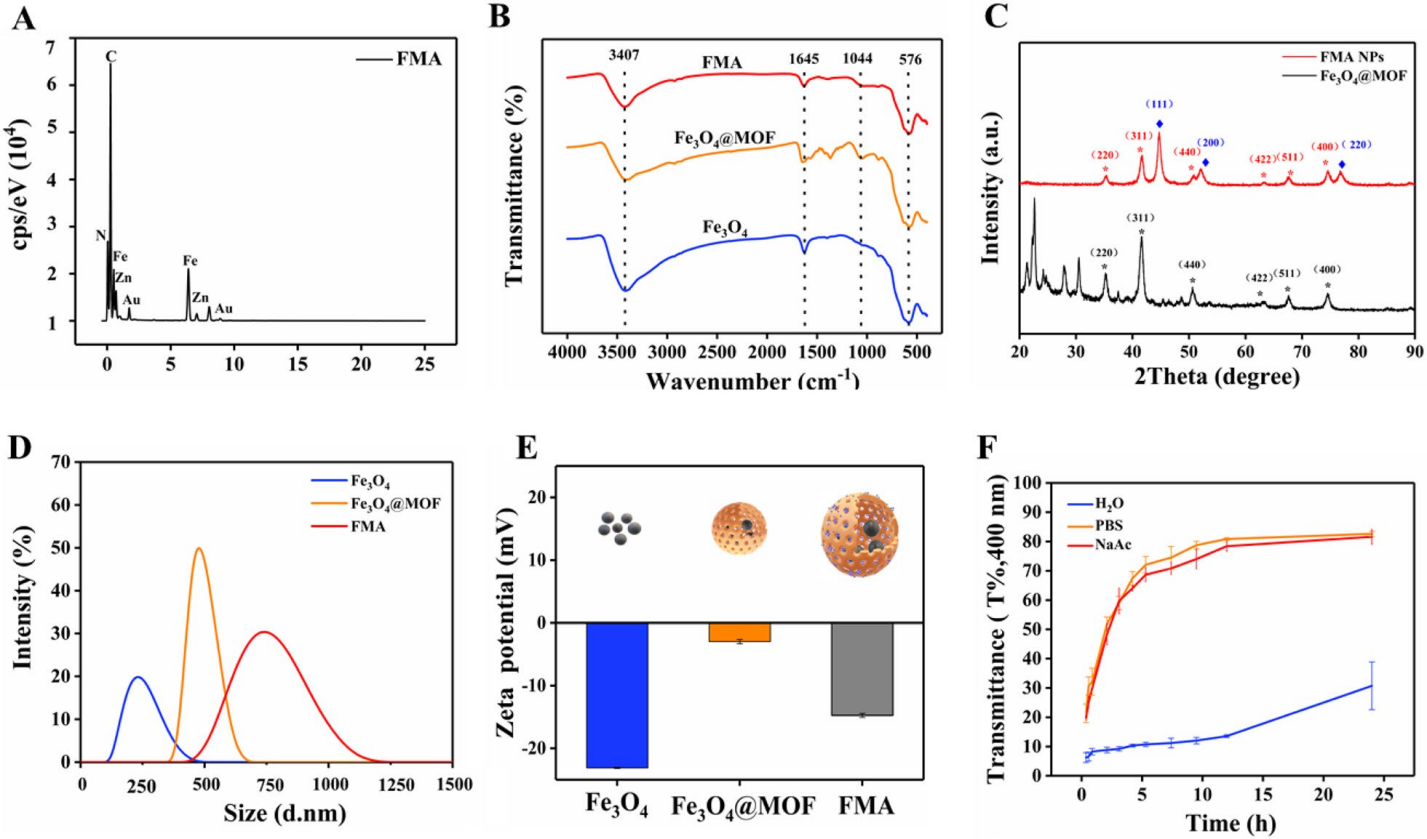
Characterisation of different nanocomposites. **A** Distribution of chemical elements of FMA NPs. **B** FTIR spectrum of Fe_3_O_4_, Fe_3_O_4_@MOF, and FMA NPs. **C** XRD patterns of Fe_3_O_4_@MOF and FMA NPs. **D** Size distribution of hydrated particles. **E** Zeta potential assay of the prepared Fe_3_O_4_, Fe_3_O_4_@MOF, and FMA NPs. **F** Analysis of stability of FMA NPs in various media including DI water, PBS solution (0.01 mol L^−1^, pH 7.4), and NaAc-HAc buffer (0.02 mol L^−1^, pH 3.6) by DLS

The as prepared nanocomposites were characterised as follows. The morphological patterns of the prepared Fe_3_O_4_ NPs, Fe_3_O_4_@MOF NPs and FMA NPs were characterised by transmission electron microscopy (TEM). TEM image reveals a spherical or elliptic structure of Fe_3_O_4_ NPs with a diameter of around 10 nm (Additional file 1: Fig. S1A). The Fe_3_O_4_@MOF NPs showed uniform spherical or more regular squares (Additional file 1: Fig. S1B). The schematic diagram of FMA NPs is shown in Fig. 1A. As shown in Fig. 1B and C, the TEM images of FMA NPs display a core-shell structure with an average particle size about 30 nm. The ultrasmall and dispersed AuNPs are effectively wrapped into pore channels of IRMOF-3 shells, thus improving their catalytic performance. Elemental mapping analysis was used to accurately assess the elemental composition of the catalytic FMA NPs. The results suggest that Au Fe, O, and Zn co-exist with FMA NPs in the energy spectrum range (Fig. 1D-I). The uniform distribution of Au in FMA NPs was verified by EDS elemental mapping (Fig. 2A).

The composition of the fabricated various nanocomposites was analysed via Fourier-transform infrared spectroscopy (FTIR). The characteristic Fe-O-Fe spectral band appeared at 576 cm^−1^ due to IRMOF-3-coated Fe_3_O_4_ NPs. The absorption peak of C-O single bond was detected at 1044 cm^−1^. The N-H stretching vibration peak at 3407 cm^−1^ corresponds to the typical amino functional group of IRMOF-3, indicating successful coating of IRMOF-3 on Fe_3_O_4_ NPs (Fig. 2B). Following anchoring of Au NPs into the pore space, a distinct absorption peak C-N of Fe_3_O_4_@MOF NPs appeared at 1645 cm^−1^, indicating intact chemical structure of IRMOF-3-modified Fe_3_O_4_ NPs. The results suggested successful fabrication of FMA NPs as expected.

To precisely analyse the composition of Fe_3_O_4_@MOF and FMA NPs, the crystal structures of the complexes were analysed via X-ray diffraction (XRD). The diffraction peaks at 2θ = 35.2°, 41.5°, 50.6°, 63.0°, 67.3°, and 74.2° correspond to the (220), (311), (400), (422), (511), and (440) crystal planes, respectively, of the Fe_3_O_4_ NPs (JCPDS 65-3107) (Fig. 2C). The diffraction peaks of MOF match the X-ray diffraction spectra reported in relevant studies previously [46, 47], indicating that the formation of the MOF shell layer did not change the crystal structure of the Fe_3_O_4_ NP. After the in situ formation of Au NPs no MOF peaks were detected in the core-shell structure of the final carrier. The crystallinity of MOF particles depends on the solvent level in the inner pores [48, 49]. Therefore, heating of the thinner MOF shell layer during the synthesis induces amorphous transition of the crystalline MOF shell layer. However, the FMA NPs 2θ = 44.7°, 52.1°, and 76.8° strongly correspond to the crystalline planes of (111), (200), (220) of the standard card of Au NPs (JCPDS 04-0784), respectively. They are also strongly consistent with the characteristic diffraction peaks of Fe_3_O_4_ NPs, suggesting that Au NPs are strongly bound to the IRMOF-3 shells. These results demonstrate the successful fabrication of FMA NPs.

The hydration particle size distribution of various nanoparticles was analysed with a dynamic laser scattering (DLS) in DI water. As shown in Fig. 2D, Fe_3_O_4_ NPs exhibit a broad particle size distribution with an average hydration particle of 320 nm, while the average hydration particle of Fe_3_O_4_@MOF NPs is 450 nm. Loading of Au NPs increases the hydration particle size of FMA NPs to about 600 nm. The hydration particle size of Fe_3_O_4_ NPs, Fe_3_O_4_@MOF NPs, and FMA NPs attended to increase sequentially, indicating successful coating of the various modified layers. The analysis of zeta potential showed that the charges of Fe_3_O_4_, Fe_3_O_4_@MOF, and FMA NPs were approximately − 23.1, -2.97 and − 14.7, respectively (Fig. 2E). The overall results indicate the successful modification of different coating layers. In addition, the stability of FMA NPs in different media at different time points was investigated. As shown in Fig. 2F, the FMA NPs showed the best stability in various media after 24 h treatment in DI. The stability of FMA NPs in PBS buffer was the worst, which may be attributed to the presence of abundant salt ions in the buffer. Neutralisation of the surface charge of the nanocomposites resulted in aggregation or precipitation of large particles. The stability of FMA NPs in NaAc-HAc buffer solution was intermediate. However, it was excellent after 10 h of experiment. Therefore, the NaAc-HAc solution was selected for further experiments.

### Evaluation of intrinsic POD-mimicking activities of FMA NPs and detection of ·OH generation

**Fig. 3:**
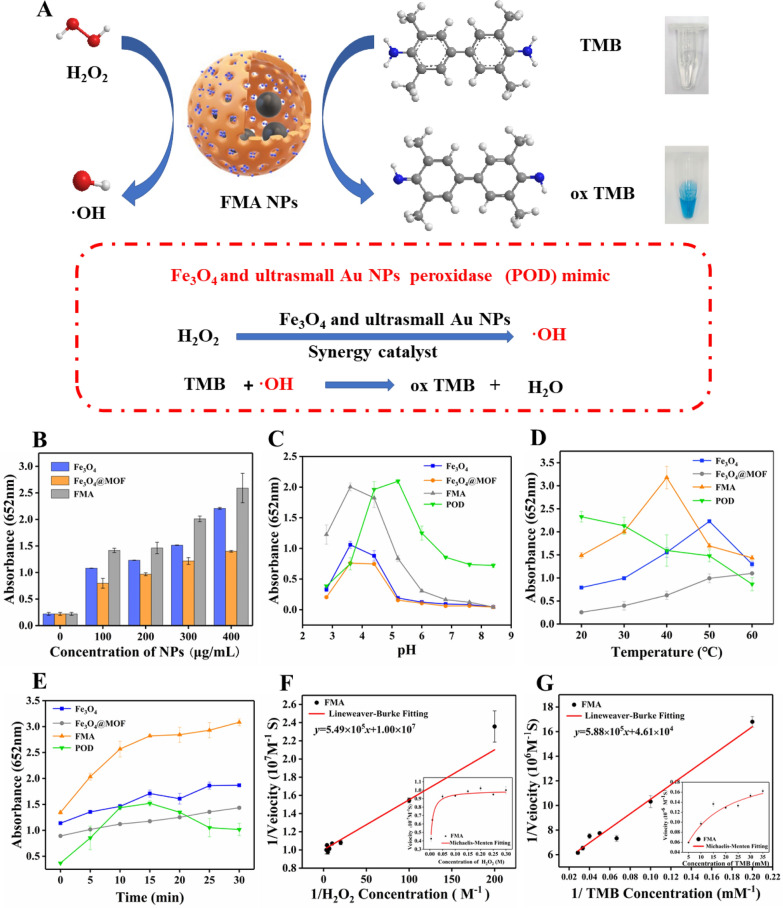
Analysis of the POD-mimicking catalytic activities and the steady-state kinetics. **A** Schematic illustrating the mechanism of enzymatic catalysis. **B** Catalytic activities of different nanoparticles with concentrations ranging from 100 µg/mL to 400 µg/mL. **C** Catalytic performance of various nanoparticles at varying levels of reaction pH. **D** Catalytic activities of different nanoparticles at varying incubation temperatures. **E** Catalytic performance of different nanoparticles at varying reaction times. **F** and **G** Line weaver-Burk plots of the initial reaction rate versus the inverse of the substrate concentration used to detect the kinetic parameters *K*_*m*_ and *V*_*max*_ of FMA NPs with TMB or H_2_O_2_ as substrate

Figure 3 A illustrates the catalytic mechanism of FMA NPs. The FMA NPs cleaved H_2_O_2_ into•OH radicals. The combination of Fe_3_O_4_ NPs and ultrasmall Au NPs displayed a synergistic POD-like activity. The colourless TMB was oxidised in the presence of •OH, resulting in a blue color. Compared with the catalytic activity of single Fe_3_O_4_ NPs or ultrasmall Au NPs, the addition of Au NPs significantly increases the catalytic activity synergistically Additional file 1: Fig. S2 shows the absorption peaks of various formulations at 652 nm. Groups without catalysts including TMB + NaAc and TMB + H_2_O_2_ showed a weak absorption peak, while other reaction systems with different catalysts exhibited a strong absorption peak at 652 nm. In addition, the simulated enzyme activity of Fe_3_O_4_@MOF NPs was slightly below that of the naked Fe_3_O_4_ NPs, which can be attributed to the adsorption of MOF on the surface of Fe_3_O_4_ NPs, leading to the reduction of catalytic centre. However, the catalytic ability of FMA NPs was significantly enhanced by the Au NPs incorporated in situ, which was attributed to the synergistic effects of the combination of Fe_3_O_4_ NPs and Au NPs. The simulated enzymatic activities of catalysts at different concentrations were also compared. The POD-mimicking activities of various catalysts increased with increasing concentration, and the absorbance of FMA NPs was the highest, indicating the strongest POD activities (Fig. 3B).

DMPO was used to determine the generation of radical species by trapping short-lived radicals to generate long-lived radical DMPO adducts, which were tested using ESR spectroscopy. The addition of hydrogen peroxide to the FMA NPs solution induced the generation of large amounts of ·OH, which was confirmed by the specific ESR spectral peaks. As shown in Additional file 1: Fig. S3, the characteristic spectrum of BMPO·OH was quadrilinear with an intensity ratio of approximately 1:2:2:1. In contrast, no signal was observed in the absence of added FMA NPs. The FMA NPs were effective in inducing the generation of ·OH radicals from H_2_O_2_ under acidic conditions.

The catalytic performance of enzymes is influenced by the reaction time, temperature, and pH. Therefore, the catalytic activities of different nanocomposites and natural POD were determined under changing reaction conditions. As shown in Fig. 3C, the FMA NPs exhibited stable catalytic activities within a broad pH range (from 3.0 to 6.5), and the optimum reaction pH was 3.6. Figure 3D shows that FMA NPs exhibit high catalytic activities between 20 and 50 °C, which overcomes the disadvantages of the natural temperature-sensitive POD. As shown in Fig. 3E, FMA NPs display significantly higher catalytic activities during the 30 min reaction time compared with the other groups, which is attributed to the synergistic catalytic effects of the combination of AuNPs and Fe_3_O_4_ NPs. However, the activities of natural POD decreased after 15 min of reaction time. Thus, compared with natural POD, FMA NPs displayed high and stable POD-mimicking activities over a wide range of pH conditions, incubation temperatures, and reaction times. The optimal reaction conditions of FSC nanozymes were pH 3.6, reaction temperature 37 °C, and a reaction time of 15 min.

### Steady-state kinetics

The kinetic parameters of the hybrid FMA nanozymes were determined under varying TMB or H_2_O_2_ concentrations and optimal reaction conditions. The results showed that the catalytic reaction induced by FMA NPs fitted the typical Michaelis-Menten model (Fig. 3F and G). Thus, we used this model to evaluate the catalytic ability of FMA nanozymes. The related kinetic parameters (*K*_m_ and *V*_max_) were derived from the Lineweaver-Burk plot. The *K*_m_ values were 0.34 and 0.24 mM for TMB and H_2_O_2_, respectively, based on Lineweaver-Burk double reciprocal plot (Fig. 3F and G). Compared with other catalysts (Additional file 1: Table S1), FMA nanozymes showed a larger *V*_max_ indicating excellent catalytic efficiency. Under similar catalyst concentrations, the value of the catalytic constant *K*_cat_ (*V*_max_ / *[E]*, in which *[E]* is the concentration of nanozyme or HRP) of FMA NPs was the largest, which indicates the highest catalytic efficiency per nanoparticle.

### Evaluation of in vitro biosafety

**Fig. 4:**
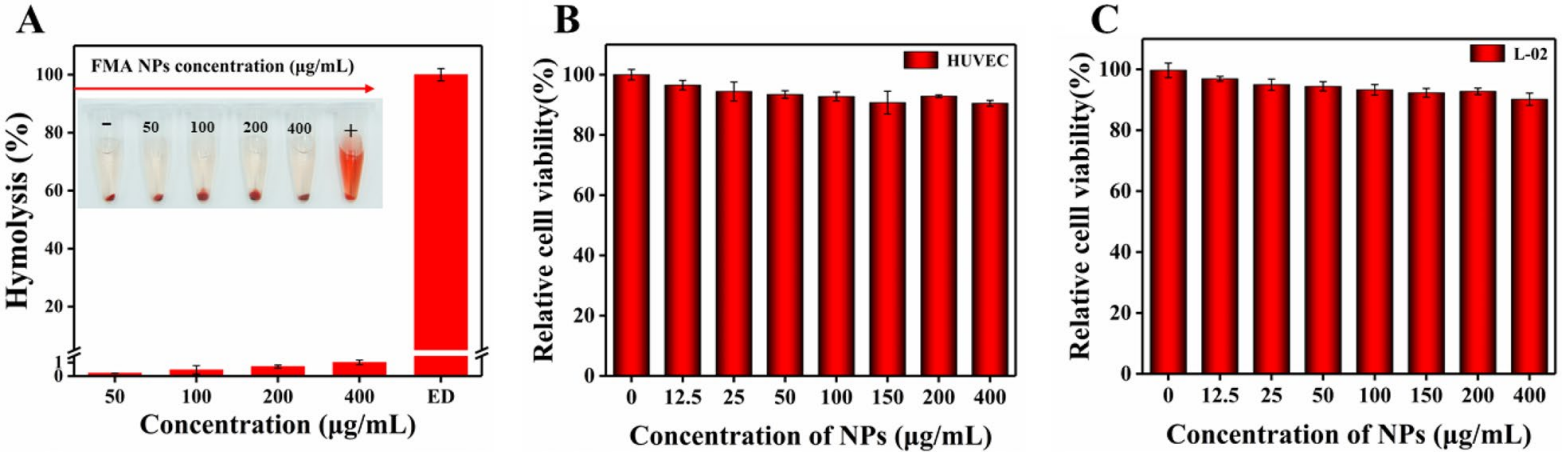
** A** The hemolytic rate of FMA NP-treated RBCs. **B** Viability of HUVECs treated with various concentrations of FMA NPs. **C** Viability of hepatocytes L-02 cells treated with various concentrations of FMA NPs

Nano-biomaterials exhibit excellent biocompatibility for clinical application, and blood compatibility is an important indicator of biocompatibility [50]. Therefore, we investigated the hemolysis of FMA NPs. As shown in Fig. 4A, rare hemolytic effects were observed under concentrations of 0-400 µg/mL. The hemolytic rate was well below 5%, indicating superior hemocompatibility at the given concentrations facilitating the antimicrobial application of FMA NPs.

HUVECs and normal hepatocytes L-02 cells were used to determine the in vitro cytotoxicity of FMA NPs. As shown in Fig. 4B and C, after treatment with FMA NPs for 24 h, the cell viabilities of HUVECs and normal hepatocytes L-02 cells were greater than 95% at low concentrations. The cell viabilities of HUVECs and hepatocytes L-02 cells remained above 90% even at FMA NP concentrations of 400 µg/mL, indicating the high biosafety of FMA NPs. Therefore, the fabricated FMA NPs displayed excellent biocompatibility and negligible toxicity.

**Fig. 5:**
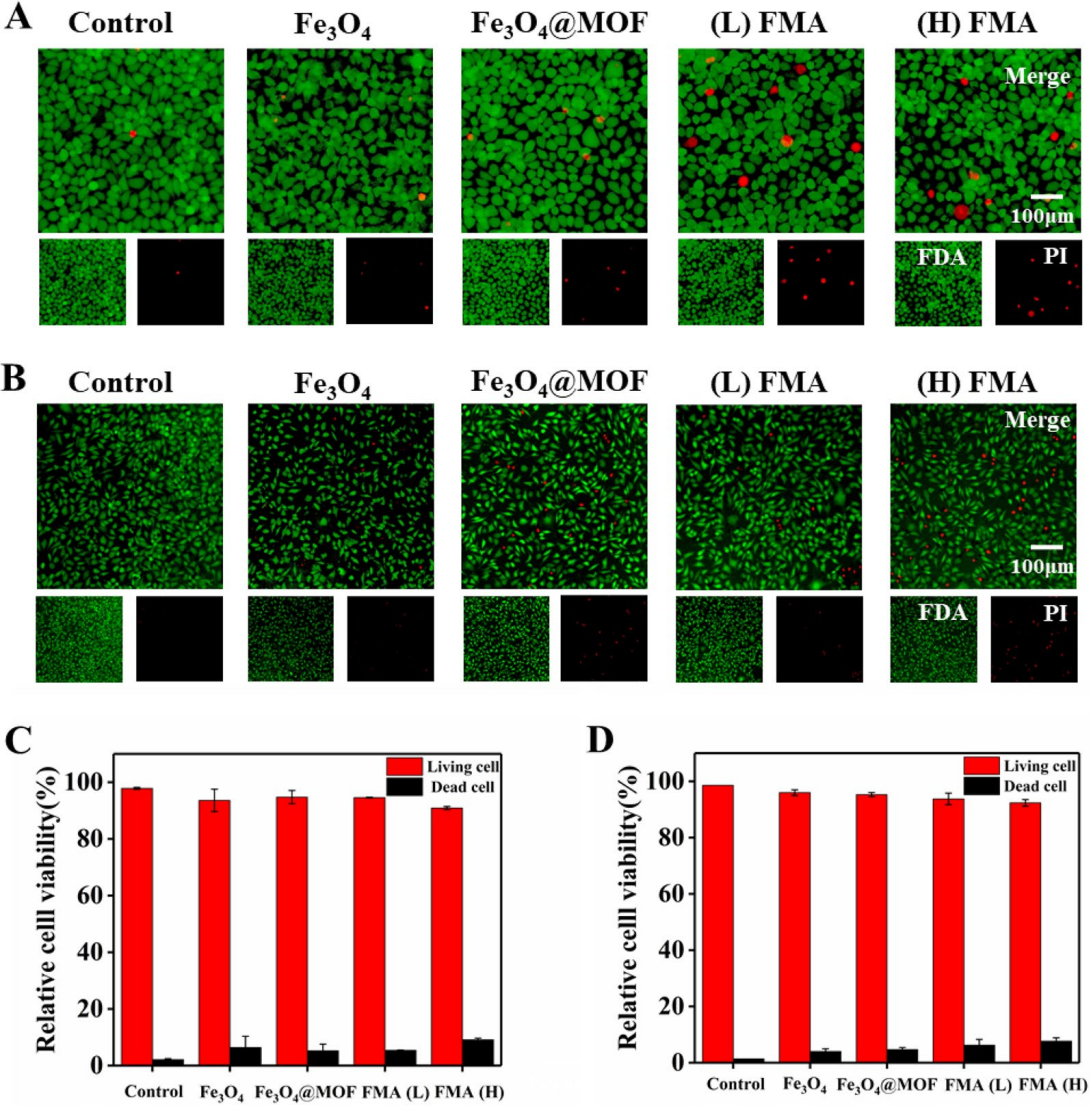
Analysis of the cell viability using FDA/PI double labelling. **A** Staining of live/dead hepatocytes L-02 cells co-cultured with different formulations. **B** Staining of live/dead HUVECs co-cultured with different formulations. **C** Analysis of relative viability of L-02 cells. **D** Analysis of relative viability of HUVECs

We also used fluorescence FDA/PI double labelling to assess the viability of L-02 cells and HUVECs after treatment with FMA NPs. The living cells were stained by FDA, and showed green fluorescence, while the dead cells were stained by PI and displayed red fluorescence [51]. As shown in Fig. 5A-B, compared with control, a small number of cells were dead after the co-incubation with different nanoparticles for 12 h. However, the percentage of both live cells was around 95% at 200 µg/mL of each nanoparticle. The viabilities of HUVECs and hepatocytes (L-02) were 91% and 92%, respectively, even in the presence of 400 µg/mL of FMA NPs (Fig. 5C and D). These results indicated that the fabricated nanoparticles displayed negligible toxicity.

### Analysis of antibacterial activity in vitro

**Fig. 6:**
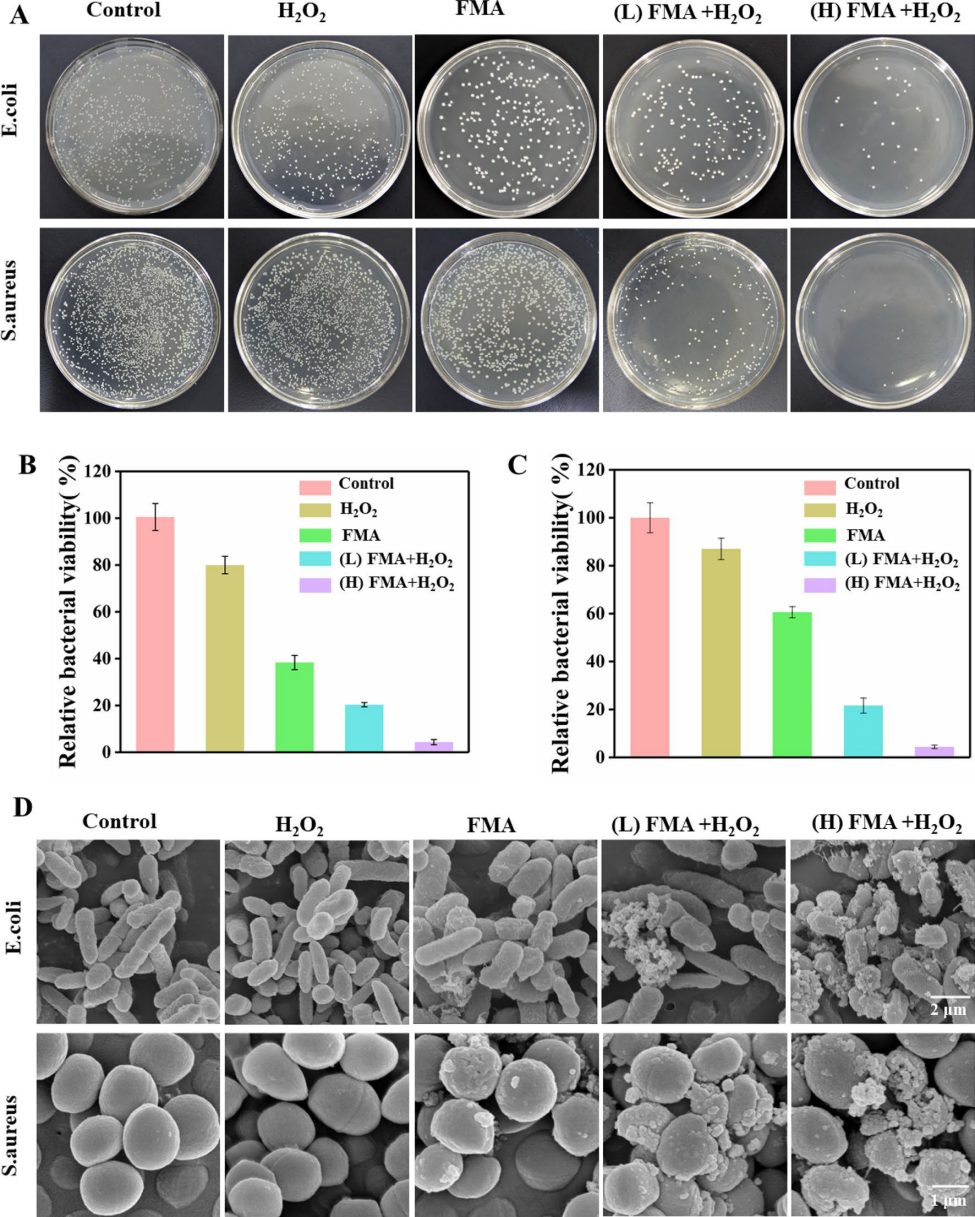
Assay of antibacterial ability of FMA NPs *in vitro.***A** Colonies of *E. coli* and *S. aureus* obtained after treatment with H_2_O_2_, FMA NPs, (low dose) FMA NPs + H_2_O_2_, and (high dose) FMA NPs + H_2_O_2_ are shown. **B** Relative activity of *E. coli* in groups treated with different agents. **C** Relative activity of *S. aureus* in different treatment groups. **D** Typical SEM analysis of *E. coli* and *S. aureus* exposed to various treatments

The antibacterial performance of FMA NPs was measured in vitro using the plate counting method. As shown in Fig. 6A, a slight decrease was observed in the number of CFU following treatment with only FMA NPs or H_2_O_2_ compared with the control, indicating that FMA NPs or H_2_O_2_ did not have a significant effect on bacterial viability. In contrast, the treatment with FMA NPs and low-dose H_2_O_2_ had a remarkable inhibiting effect on bacterial growth. The antibacterial rates were 76% and 72% for *E. coli* and *S. aureus* at low concentrations (200 µg/mL) of FMA NPs and H_2_O_2_, respectively. Especially, the antibacterial activities of *E. coli* and *S. aureus* reached 97% and 98% following treatment with 400 µg/mL FMA NPs and H_2_O_2_, which was attributed to the production of abundant toxic·OH radicals. As shown in Additional file 1: Fig. S4, the MIC values suggested that treatment with the combination of FMA NPs and H_2_O_2_ resulted in increased inhibition against *E. coli* and *S. aureus.* The MIC was achieved by increasing the concentration of FMANPs to 400 µg/mL, and when the concentration reached 500 µg/mL, the inhibition of the two bacteria was greater than 99%.

### Morphological changes in bacterial species after treatment

The morphological changes of *E. coli* and *S. aureus* treated with different formulations were determined by SEM. The untreated *E. coli* and *S. aureus* showed rod-like and spherical morphology with smooth surface, respectively. When treated with H_2_O_2_, *E. coli* and *S. aureus* rarely displayed significant changes. Treatment with FMA NPs led to insignificant changes in the overall bacterial morphology, indicating a slight antibacterial activity against *E. coli* and *S. aureus*. Co-treatment with high levels of FMA NPs and H_2_O_2_ disrupted bacterial integrity and destroyed the bacterial morphology with unclear borders between bacteria, different degrees of surface depression and dents due to the generation of toxic ·OH (Fig. 6D). These results were consistent with the colony-forming units, which were further confirmed by the improved antibacterial effects of FMA NPs and H_2_O_2_.

### Biofilm clearance

**Fig. 7:**
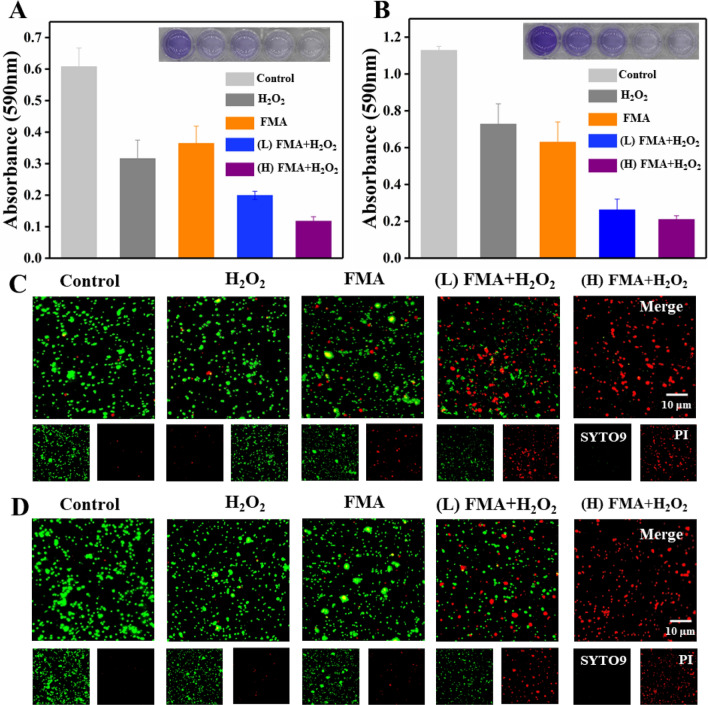
** A** Disruption of *E. coli* biofilms in various treatment groups. **B** Biofilm disruption effect on *S. aureus* in various treatment groups. **C** Fluorescence images of live/dead *E. coli* (green/red) with different treatments. **D** Fluorescence images of live/dead *S. aureus* (green/red) under different treatments

Biofilm formation was to analysed to determine the effects of various formulations using crystalline violet staining. The biofilm was slightly removed from the bottom of the 24-well microplates following treatment with FMA NPs or H_2_O_2_, while the treatment with the combination of FMA NPs and H_2_O_2_ led to significant biofilm removal. As shown in Fig. 7A and B, the treatment with high concentrations of FMA NPs (400 µg/mL) and H_2_O_2_ yielded the strongest anti-biofilm effects, and the biofilm inhibition rate of *E. coli* and *S. aureus* reached 83% and 91%, respectively. We also investigated the ring-breaking ability on biofilms by incubating different formulations with pre-formed biofilms. Compared with control biofilms, which were barely cleared by treatment with H_2_O_2_ and FMA NPs, the inhibition of *E. coli* and *S. aureus* reached 75% and 85% following treatment with FMA NPs and H_2_O_2_ (Additional file 1: Fig. S5A and B). These results also demonstrated obvious enhancement in the antibacterial activities of FMA NPs and H_2_O_2_.

### Analysis of live and dead bacteria

To further investigate the enhanced antibacterial abilities of FMA NPs, nucleic acid dyes SYTO9 and PI were employed in live/dead cell assays. As shown in Fig. 7C and D, the green fluorescence signals of *E. coli* and *S. aureus* were predominant in the control group, which indicated that almost no dead bacteria. Treatments with H_2_O_2_ or FMA NPs revealed limited red fluorescence due to the low antibacterial efficiency. By contrast, co-treatment with H_2_O_2_ and FMA NPs significantly increased the number of dead bacteria due to the production of highly toxic ·OH radicals as shown by the predominant red fluorescence signal, which suggested that the antibacterial efficiency was greatly enhanced and resulted in extensive bacterial death (Fig. 7C and D). Exposure to high concentrations of FMA NPs and H_2_O_2_ led to the disappearance of the green fluorescence signal, indicating that the bactericidal ability depended on the concentration of FMA NPs.

### Evaluation of wound-healing effects

**Fig. 8:**
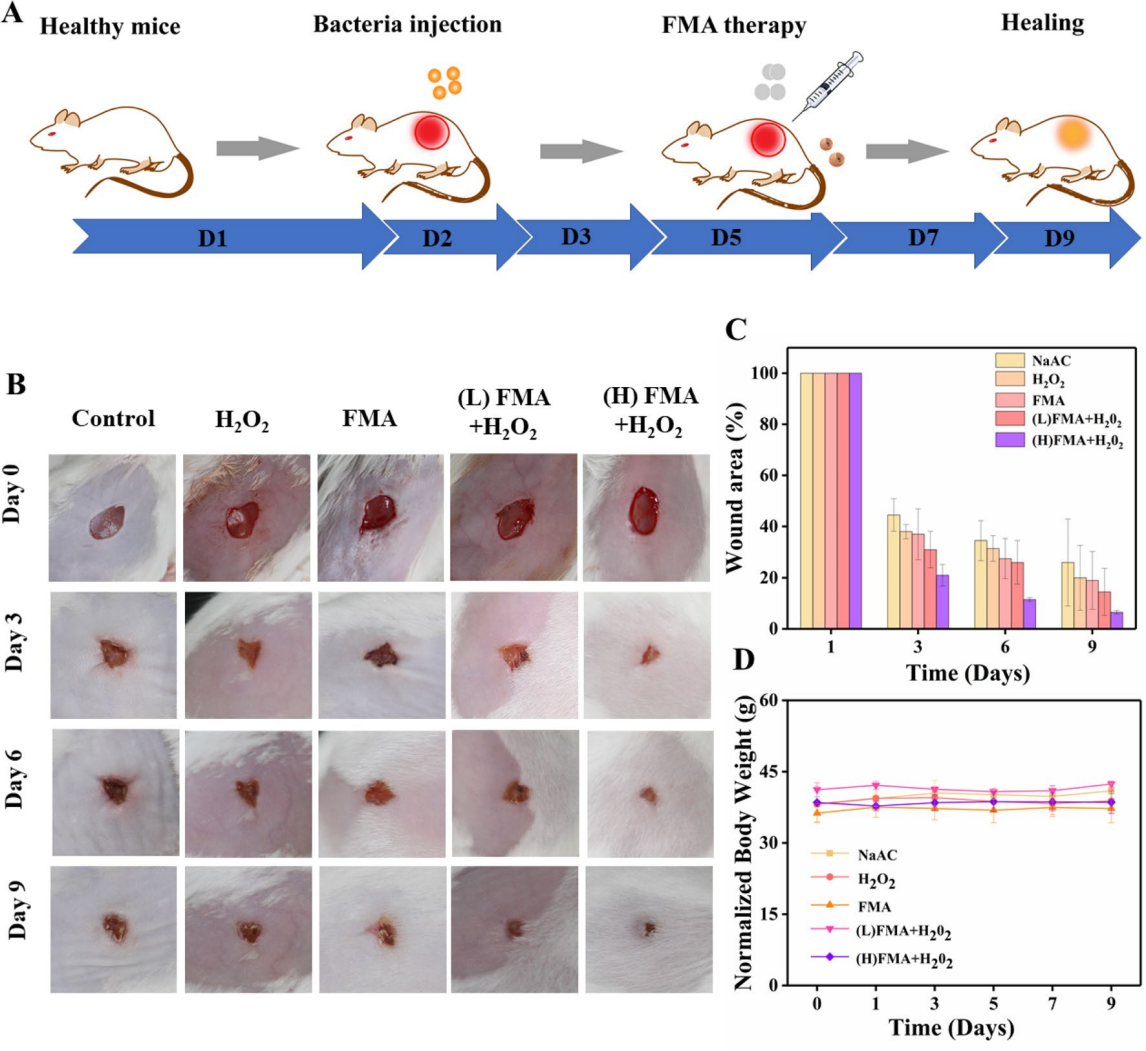
** A** Model diagram of mouse treatment. **B*** S. aureus*-infected wounds on the back of mice under various treatments. **C** Relative wound area in different mice after 8 days. **D** Changes in mouse body weight under various treatments during the therapeutic period

Given the excellent antibacterial effects observed, the potential wound-healing effects of FMA NPS in vivo were investigated for KM mice. Five groups of random mouse models were developed by generating dorsal skin wounds. Subsequently, bacterial wound infections were created by exposing the wounds to 10^7^ CFU/mL *S. aureus.* Various formulations were injected into the wound after 1-day infection. Figure 8A shows the treatment protocol in the animal model. As shown in Fig. 8B, a relatively slow wound healing was detected in the control, and after treatment with H_2_O_2_ or FMA NPs, which was attributed to the low catalytic ability. By contrast, the wound healing rate reached more than 80% by day 3 post-injection when treated with FMA NPs and H_2_O_2_. The wound crusted and completed healed after 9 days post-injection, and the healing rate was remarkably higher than in the other groups (Fig. 8C), indicating the wound-healing effects of ·OH radicals. Significantly, the treatment with FMA NPs and H_2_O_2_ significantly accelerated the healing process compared with the control. More importantly, the body weight of the treated mice did not change obviously, indicating the absence of any toxic side effects associated the designed FMA nanozymes in vivo (Fig. 8D).

**Fig. 9:**
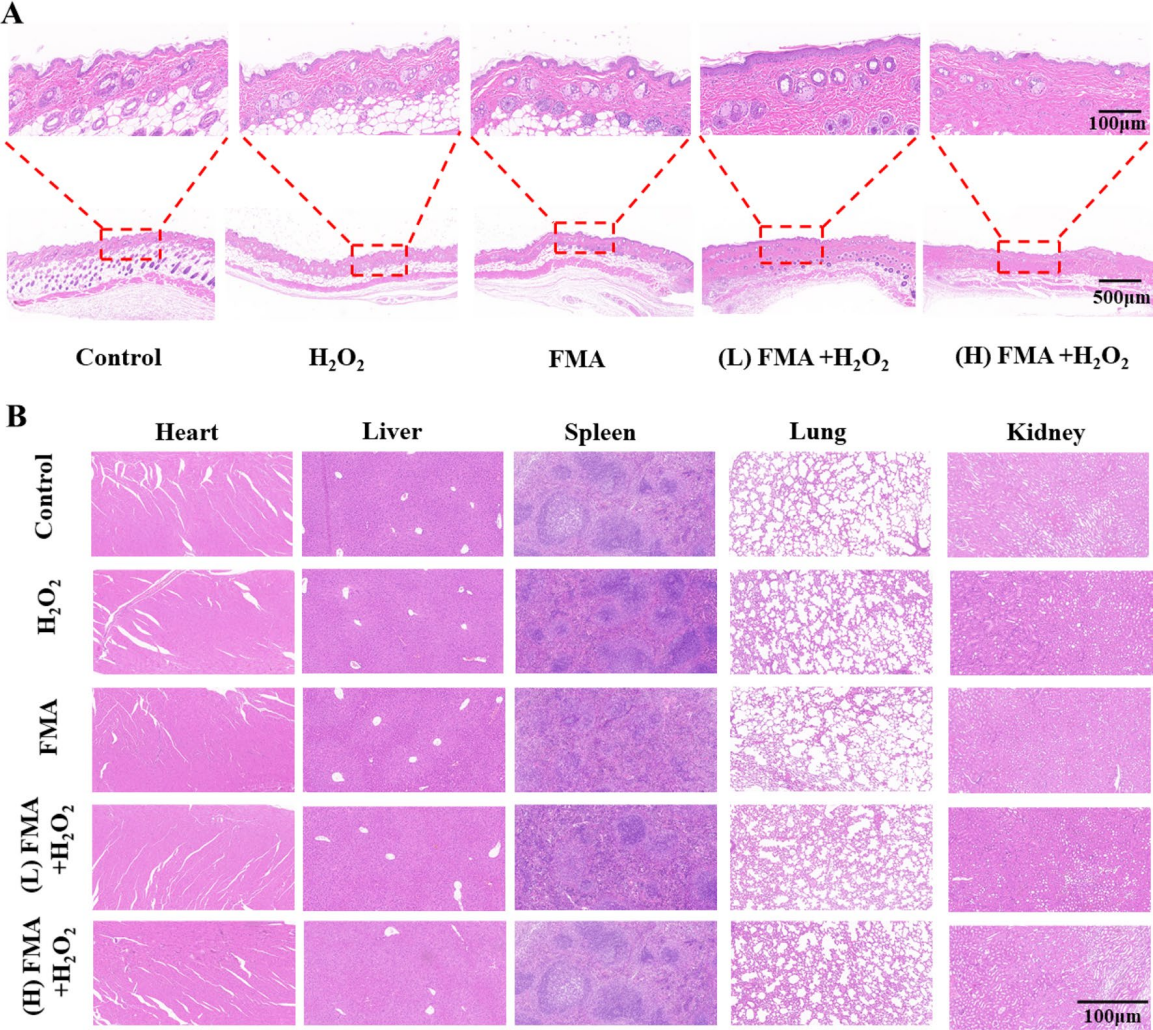
H&E staining histological analysis after 8 days of treatment. **A** Skin tissues. **B** Key organs including heart, liver, spleen, lungs, kidneys, and skin

To assess the wound-healing efficiency in various mice, hematoxylin and eosin (H&E) staining was performed for histological analysis of the wound tissues. As shown in Fig. 9A, compared with decreased wound closure with incomplete epidermal layers and inflammatory cells observed after H_2_O_2_ treatment, an intact epidermal layer in the wounds was observed after treatment with FMA NPs and H_2_O_2_, and the mice displayed a high degree of wound re-epithelialisation when treated with high concentrations. Thus, the prepared hybrid FMA nanozymes displayed an excellent wound-healing ability in vivo. Further, the H&E staining of the key organs in the treated mice displayed no lesions, indicating the biosafety of FMA nanozymes in vivo (Fig. 9B).

## Conclusion

In summary, we report the fabrication of an innovative bimetallic FMA hybrid nanozyme for antibacterial wound healing. The nanozymes were constructed with ultrasmall Au NPs-anchored MOFs stabilising Fe_3_O_4_ NPs. The well-designed FMA hybrid nanozymes displayed significantly enhanced POD-mimetic catalytic activities due to the synergistic effects between Au NPs and Fe_3_O_4_ NPs. The FMA nanozymes exposed to low doses of H_2_O_2_ generated a large amount of ·OH and accelerated the antibacterial efficiency in vitro and wound healing in vivo. Further, the FMA nanozymes showed negligible biotoxicity even at high concentrations. In summary, this study demonstrates the promise and potential of non-toxic nanozymes for clinical antibacterial applications.

### Supplementary Information

**Additional file 1:** **Fig. S1.** **A** TEM images of Fe_3_O_4_ NPs, **B** TEM images of Fe_3_O_4_@MOF NPs. **Fig. S2.** Analysis of the POD mimicking activities of different formulations using UV–vis spectra (a, b, c, d and e represent of TMB+H_2_O_2_+FMA NPs, TMB+H_2_O_2_+Fe_3_O_4_ NPs, TMB+H_2_O_2_+ Fe_3_O_4_@ MOF NPs, TMB+ H_2_O_2_, TMB+ NaAc). **Table S1.** Comparison of the kinetic parameters* K*_m_ and *V*_max_ of FMA NPs, HRP, and other reported materials. **Fig. S3.** The ESR spectra of FMA NPs (400μg/mL), H_2_O_2_ used as control. **Fig. S4.** **A** The determination of MIC of *E. coli*. **B** The determination of *S. aureus*. **Fig. S5. A** Inhibitory effects of FMA NPs on *E. coli* biofilm. **B** Inhibitory effects of FMA NPs on *S. aureus*).
