# Supplementary material for: Metal-organic framework-modulated Fe3O4 composite au nanoparticles for antibacterial wound healing via synergistic peroxidase-like nanozymatic catalysis
Source: J Nanobiotechnology. 2023 Nov 15;21:427. doi: 10.1186/s12951-023-02186-6 (PMC10647143; doi:10.1186/s12951-023-02186-6)
Supplement: Supplementary file 1 — Additional file 1: Fig. S1. A TEM images of Fe3O4 NPs, B TEM images of Fe3O4@MOF NPs. Fig. S2. Analysis of the POD mimicking activities of different formulations using UV–vis spectra (a, b, c, d and e represent of TMB+H2O2+FMA NPs, TMB+H2O2+Fe3O4 NPs, TMB+H2O2+ Fe3O4@ MOF NPs, TMB+ H2O2, TMB+ NaAc). Table S1. Comparison of the kinetic parameters Km and Vmax of FMA NPs, HRP, and other reported materials. Fig. S3. The ESR spectra of FMA NPs (400μg/mL), H2O2 used as control. Fig. S4. A The determination of MIC of E. coli. B The determination of S. aureus. Fig. S5. A Inhibitory effects of FMA NPs on E. coli biofilm. B Inhibitory effects of FMA NPs on S. aureus). [file 12951_2023_2186_MOESM1_ESM.doc]

**Additional Information**

**Metal-Organic Framework-Modulated Fe_3_O_4_ Composite Au Nanoparticles for Antibacterial Wound Healing via Synergistic Peroxidase-like** **Nanozymatic Catalysis**

Chuan Liu^1,2^, Xuanping Zhao^1,2^, Zichao Wang^1,2^, Yingyuan Zhao^1,2^, Ruifang Li ^1,2^, Xuyang Chen^1,2^,

Hong Chen^1,2^, Mengna Wan^1,2^, Xueqin Wang*^1,2^

^1^*College of Bioengineering, Henan University of Technology, Zhengzhou, Henan 450001, China*

^2^*Key Laboratory of Functional Molecules for Biomedical Research, Henan University of Technology, Zhengzhou, Henan 450001, China.*

**Corresponding author:*

*Xueqin Wang, Tel: + 86 371 67756928, E-mail address:*[*wangxq0708@163.com*](mailto:wangxq0708@163.com)

***Synthesis of Fe_3_O_4_ NPs***

As the magnetic nanocarriers used in the present study, the Fe_3_O_4_ NPs were prepared according to the previous methods proposed elsewhere [1,2]. First, 3.0 mL FeCl_3_ (2 mol/L dissolved in 2 mol/L HCl) was added to 10 mL of ultra-purified water and stired for 30 min, and then 2 mLNa_2_SO_3_ (1 mol/L) was subsequently added dropwise into the mixture within 5 min while stirring. When the solution color changed from red to light yellow, the solution was added into 80 mLNH_4_OH (0.85 mol/L) while vigorously stirring. A black precipitate quickly formed and was completely crystallized for another 40 min. The black precipitate was washed with deoxygenated water until the solution pH less than 7.5 and then dried to form Fe_3_O_4_ powder.

***Materials***

Chloroauric acid (HAuCl_4_), N-N dimethylformamide (DMF) was purchased from Aladdin Industrial Co., Ltd. (Shanghai, China). Natural POD (≥250 U/mg), agar powder, yeast extract, and tryptone were purchased from Solarbio Technology Co., Ltd (Beijing, China). 2-aminoterephthalic acid was purchased from Maclean's Reagent Co. Zinc nitrate hexahydrate (Zn (NO_3_)_2_-6H_2_O), and polyvinylpyrrolidone (PVP) was purchased from Dingguo Biotechnology Co. 3, 3’, 5, 5’-tetramethylbenzidine (TMB), hydrogen peroxide (H_2_O_2_), fluorescein diacetate (FDA) and propidiumiodide (PI) were obtained from Sigma-Aldrich (St. Louis, MO, USA). SYTO9/PI double- staining kit was purchased from Mao Kang Biological Co Ltd (Shanghai, China). Cell Counting Kit-8 (CCK-8) was supplied by Bayside Biotechnology Co (Shanghai, China). Crystal violet ammonium oxala (CV 2%) was purchased from Sollerbauer Chemical Reagents Ltd (Shanghai, China). Other reagents and chemicals were purchased from local commercial suppliers and were of analytical grade.

***Characterization methods***

The prepared Fe_3_O_4_ and FMA NPs were morphologically characterized by high-resolution electron microscopy (HRTEM, JEM-2100, Japan) and high-angle annular dark-field scanning electron microscopy (HAADF-STEM, Japan). The crystal structures of the Fe_3_O_4_ and FMA NPs were analyzed by X-ray diffractometer (PANalytical analyzer, The Netherlands). The infrared spectra of the various nanocomposites were recorded by Fourier transform infrared spectroscopy (FT-IR) (Nicolet IS50-Continuum, Inc., USA). The hydrated particle size distribution and zeta potentials of various nanocomposites were determined by dynamic laser scattering (DLS), and the stability of the various nanocomposites was analyzed with Malvern Zetasizer nano series (Nano ZS90). The distribution of each element of FMA NPs was analyzed by field emission transmission electron microscopy (TEI-Tecanai G2 F30, USA). The microscopic morphology of the treated bacteria was observed by biological transmission electron microscopy (JEM-1200EX, Japan). Live/dead staining of the treated bacteria was imaged under an inverted fluorescence microscope (Tis Nikon, Japan)

***Kinetic analysis***

The steady-state kinetic was analysed investigated by recording the changes of absorbance at 652 nm under optimal experimental conditions with a microplate spectrophotometer. The reaction system consisted of 20 μL FMA NPs (400 μg mL^-1^) in 400 μL NaAc-Hac buffer with various concentrations of TMB or H_2_O_2_. The absorbance values obtained were converted into concentrations of blue oxidized TMB (ox TMB) products by Beer-Lambert.


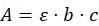


Where *A* is absorbance, *ε* is the molar absorption coefficient, *b* is the optical path, *c* is the concentration of a light-absorbing substance, and the *ε* of ox TMB at 652 nm is 3.9×10^4^ M^−1^cm^−1^ [3]. The kinetic constants (*V_max_* and *K_m_*) were calculated using the Michaelis-Menten equation as follows:


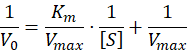


Where *V_0_* and *V_max_* are initial and maximum velocity, respectively; [*S*]is the substrate concentration, and *K_m_* is Michaelis constant [4].

***Statistical analysis***

All the data represented as mean ±standard deviation (SD). Student’s *t*-test was used for statistical comparisons. *p< 0.05 was considered significant, **p< 0.01 was considered moderately significant and ***p< 0.001 was considered highly significant.


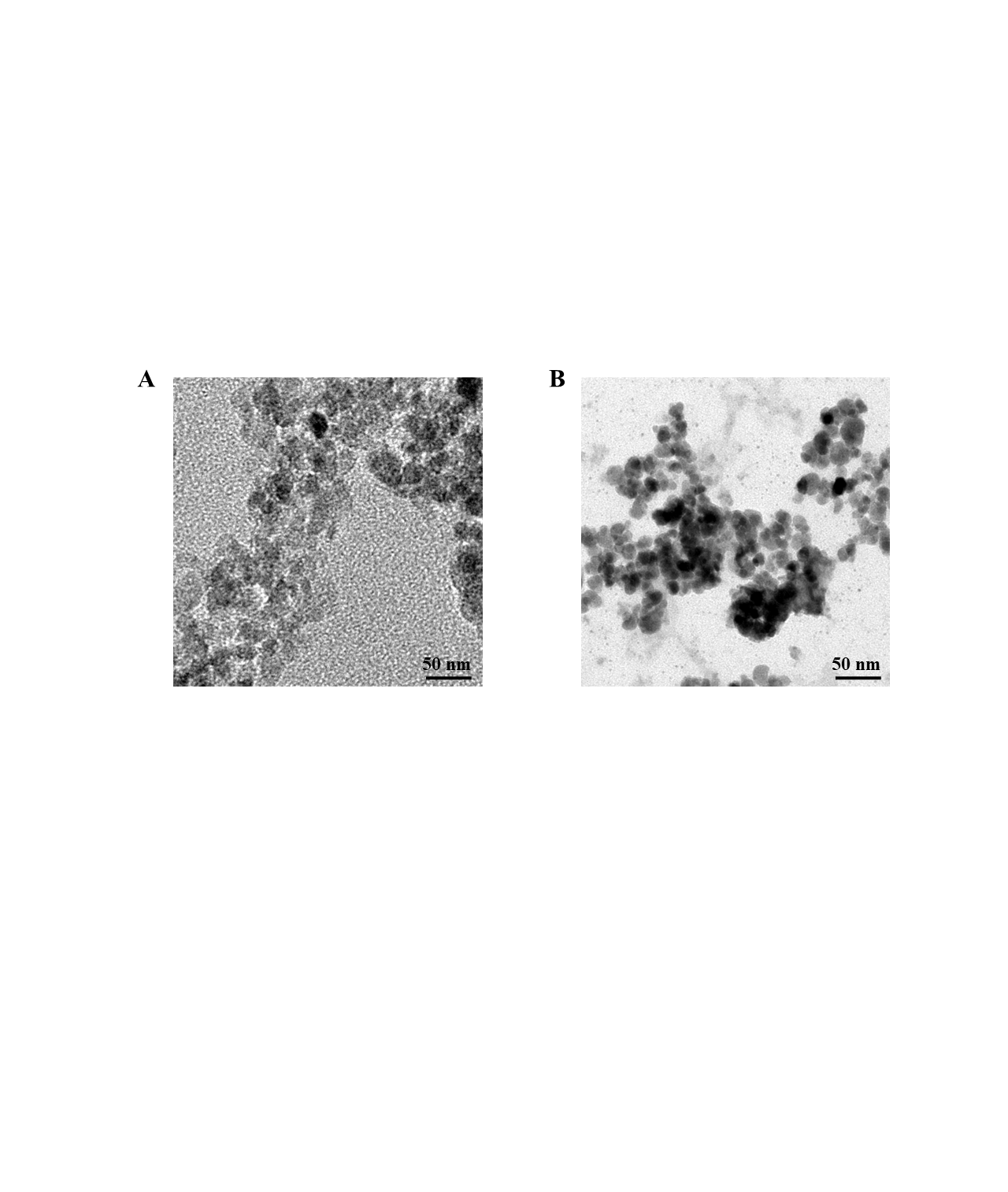


**Fig. S1** **A** TEM images of Fe_3_O_4_ NPs, **B** TEM images of Fe_3_O_4_@MOF NPs.


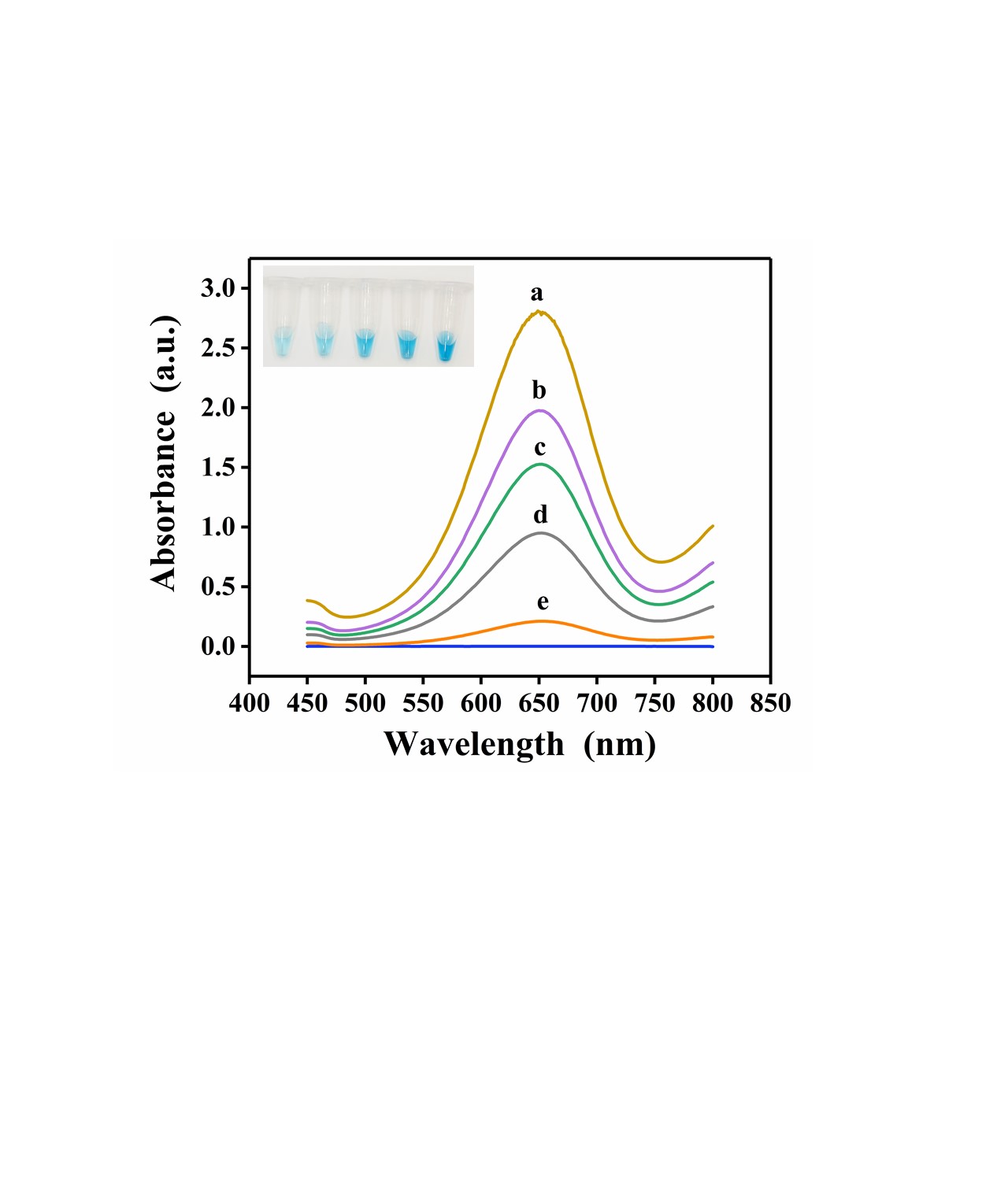


**Fig. S2** Analysis of the POD mimicking activities of different formulations using UV–vis spectra (a, b, c, d and e represent of TMB+H_2_O_2_+FMA NPs, TMB+H_2_O_2_+Fe_3_O_4_ NPs, TMB+H_2_O_2_+ Fe_3_O_4_ @ MOF NPs, TMB+ H_2_O_2_, TMB+ NaAc).

**Table. S1**

**Comparison of the kinetic parameters *K_m_* and *V_max_* of FMA NPs, HRP, and other reported materials.**

| Catalyst | Substrate | *K_m_* (mM) | *V_max_* (10^-8^ M s^-1^) | Reference |
| --- | --- | --- | --- | --- |
| MMSN/AuNPs | TMB | 15.37 | 0.91 | [5] |
|  | H_2_O_2_ | 6.35 | 3.85 |  |
| Fe_3_O_4_-TiO_2_/graphene NPs | TMB | 0.125 | 5.23 | [6] |
|  | H_2_O_2_ | 0.086 | 9.46 |  |
| TiO_2_ | TMB | 191 | 16.7 | [7] |
|  | H_2_O_2_ | 0.223 | 13.9 |  |
| HRP | TMB | 0.434 | 10 | [8] |
|  | H_2_O_2_ | 3.7 | 8.71 |  |
| FMA NPs | TMB  H_2_O_2_ | 0.34  0.24 | 23.5  10.1 | This work |


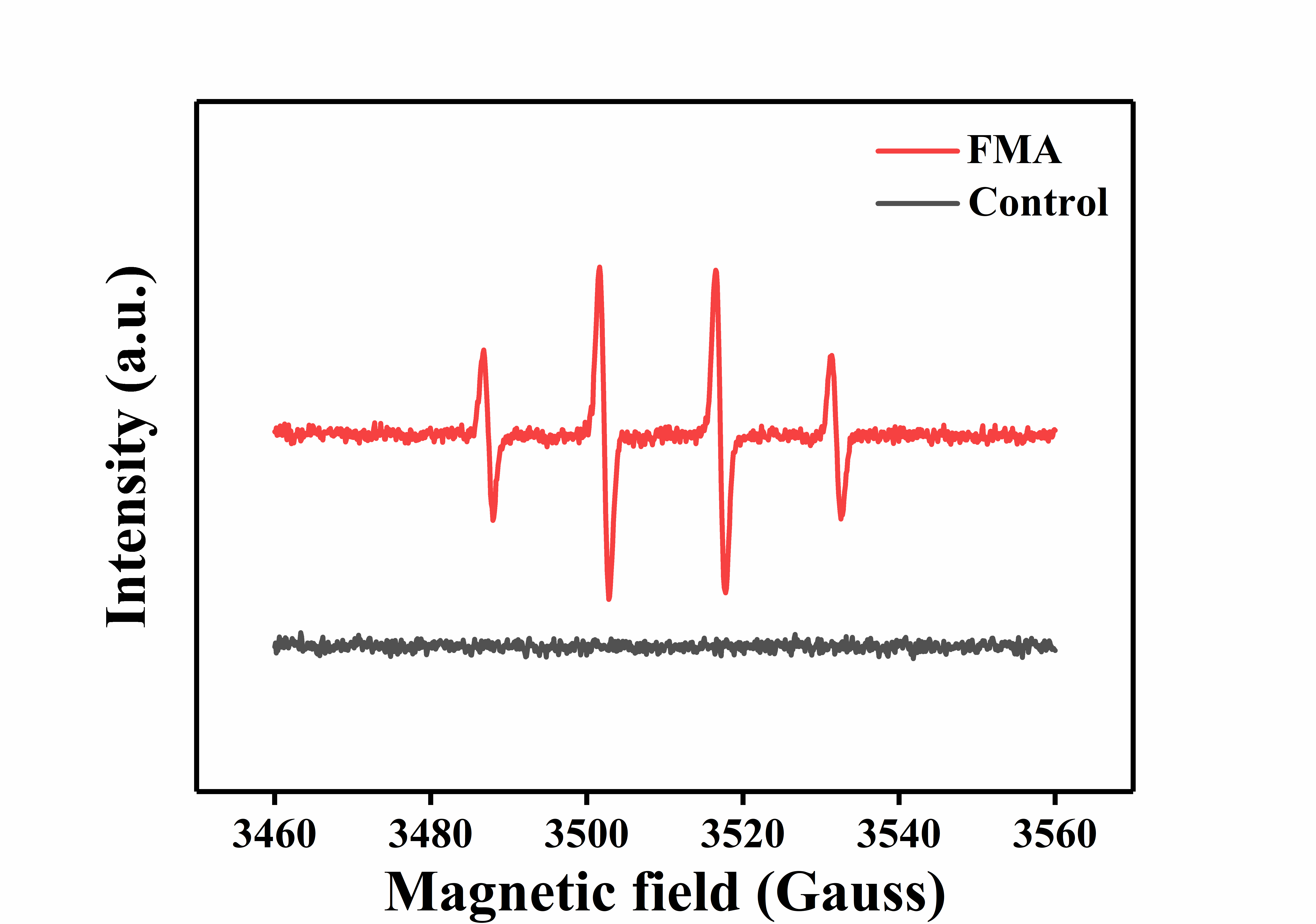


**Fig. S3** The ESR spectra of FMA NPs (400μg/mL), H_2_O_2_ used as control.


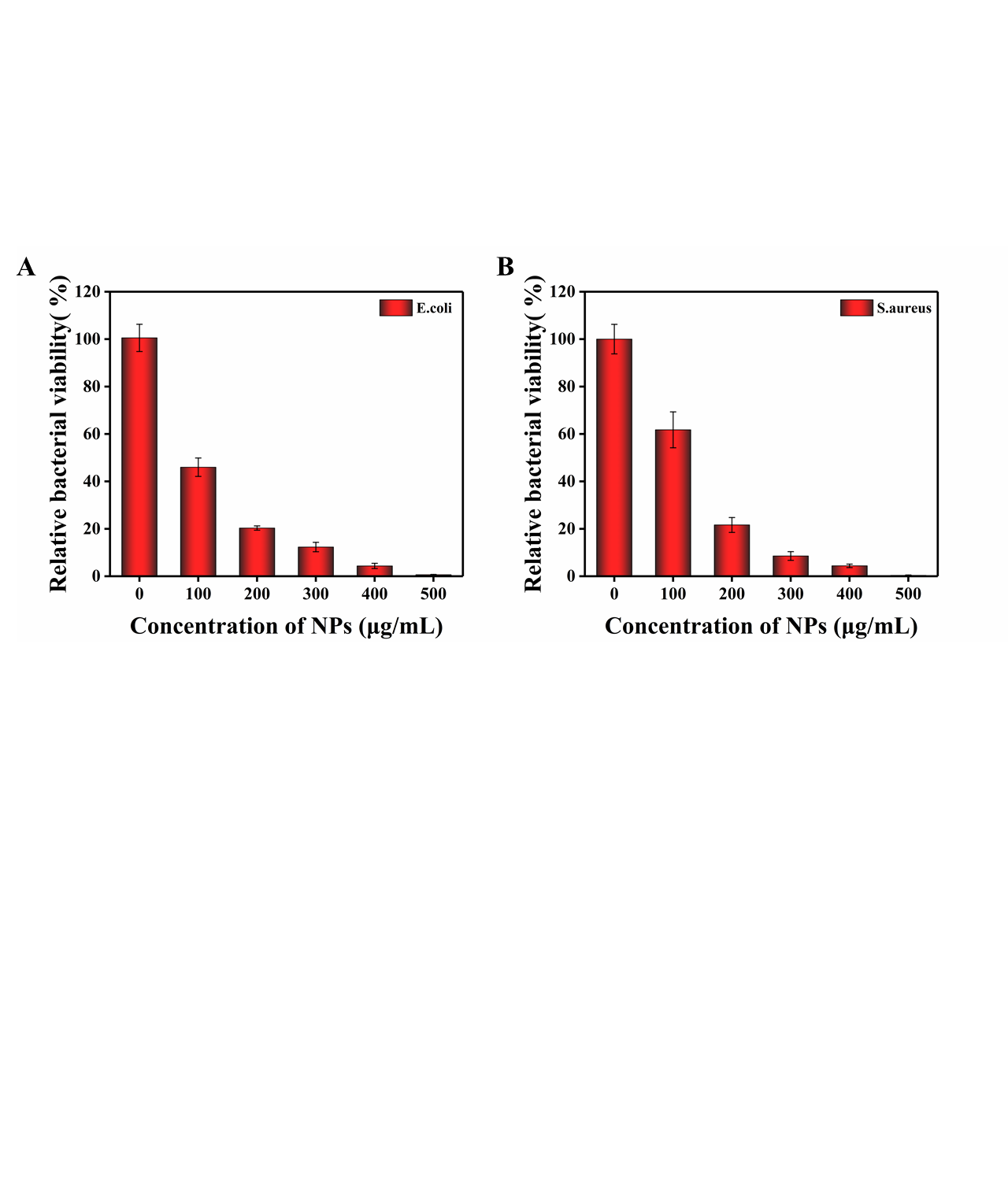


**Fig. S4 A** The determination of MIC of *E. coli*. **B** The determination of *S. aureus*.


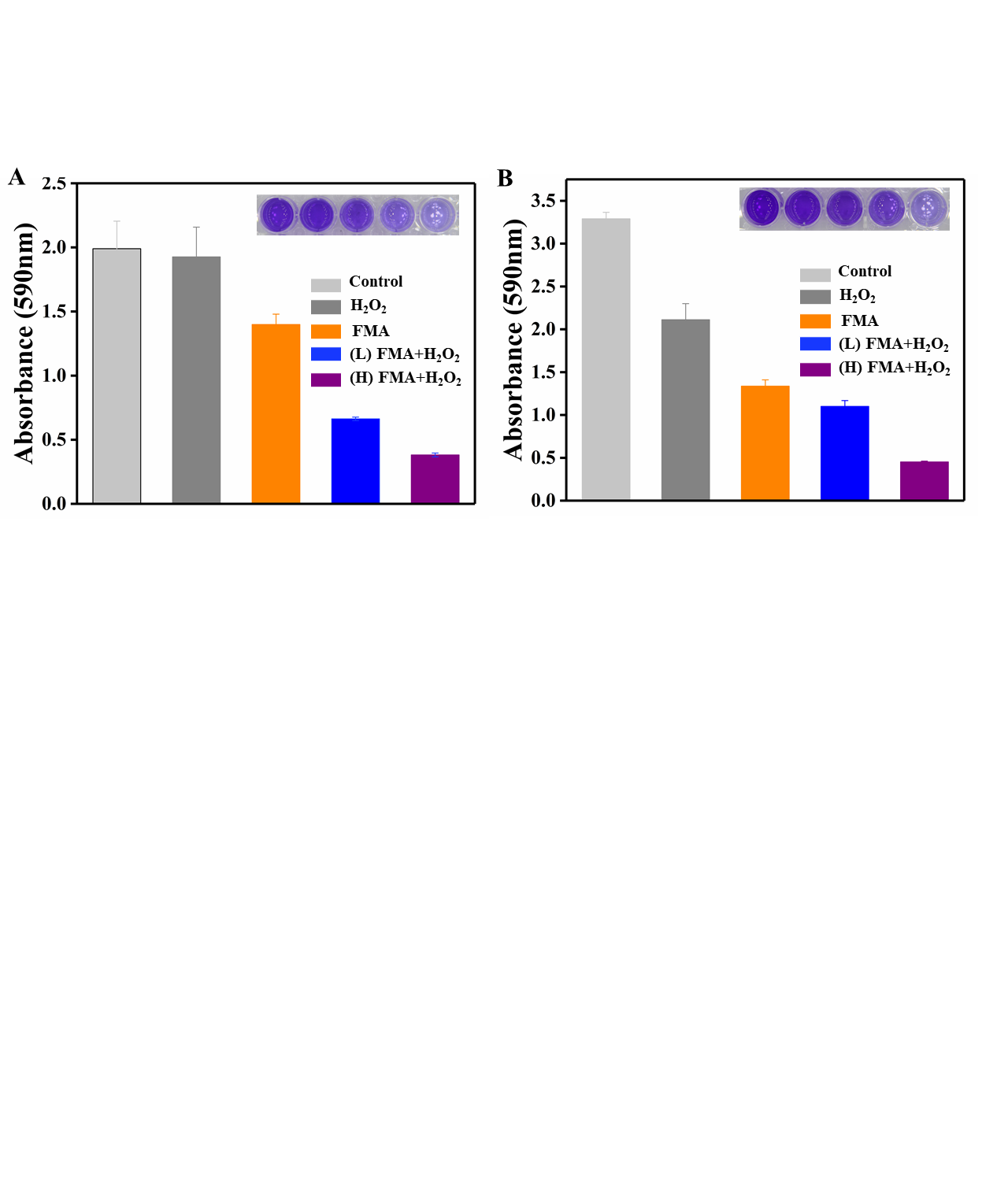


**Fig. S5 A** Inhibitory effects of FMA NPs on *E. coli* biofilm. **B** Inhibitory effects of FMA NPs on *S. aureus*).

**References**

[[1] Qu S, Yang H, Ren D, Kan S, Zou G Li D, Li M. Magnetite nanoparticles prepared by precipitation from partially reduced ferric chloride Aqueous Solutions. J. Colloid Interface Sci. 1999;215: 190–192.

[2] Sun YK, Ming M, Zhang Y, Gu N. Synthesis of nanometer-size maghemite particles from magnetite. Colloids Surf. A. 2004;245: 15–19.

[3] Zhang XQ, Gong SW, Zhang Y, Yang T, Wang CY, Gu N. Prussian blue modified iron oxide magnetic nanoparticles and their high peroxidase-like activity. J. Mater. Chem. 2010;20: 5110–5116.

[4] Cooney MJ. Kinetic measurements for enzyme immobilization. Methods Mol. Biol. 2011;679: 207–225.

[5] Wang XQ, Xiong TD, Cui MM, Guan XL, Yuan JC, Wang ZC. targeted self-activating au-fe_3_o_4_ composite nanocatalyst for enhanced precise hepatocellular carcinoma therapy via dual nanozyme-catalyzed cascade reactions. Appl. Mater. Today 2020;2.

[6] Boruah PK, Das MR. Dual Responsive Magnetic Fe_3_O_4_-TiO_2_/Graphene nanocomposite as an artificial nanozyme for the colorimetric detection and photodegradation of pesticide in an aqueous medium. J. Hazard Mater. 2020;385.

[7] Wang D, Zhang B, Ding H, Liu D, Nie G. TiO_2_ Supported single Ag atoms nanozyme for elimination of SARS-Cov2. Nano. Today 2021;40.

[8] Zhang XQ, Gong SW, Zhang Y, Yang T, Wang CY, Gu N. Prussian blue modified iron oxide magnetic nanoparticles and their high peroxidase-like activity. J. Mater. Chem. 2010;20(24): 5110-5116.
